# Coronary sinus thrombosis in a patient with amyloidosis, the role of multimodality imaging: a case report

**DOI:** 10.1093/ehjcr/ytae453

**Published:** 2024-08-26

**Authors:** Alejandro Manuel López-Pena, Rosa Alba Abellás-Sequeiros, Andrea López-López, Jeremías Bayón-Lorenzo, Carlos González-Juanatey

**Affiliations:** Cardiology Department, Hospital Universitario Lucus Augusti, 27003 Lugo, Spain; CardioHULA Research Group, Fundación Instituto de Investigación Sanitaria de Santiago de Compostela FIDIS, 27003 Lugo, Spain; Cardiology Department, Hospital Universitario Lucus Augusti, 27003 Lugo, Spain; CardioHULA Research Group, Fundación Instituto de Investigación Sanitaria de Santiago de Compostela FIDIS, 27003 Lugo, Spain; Cardiology Department, Hospital Universitario Lucus Augusti, 27003 Lugo, Spain; CardioHULA Research Group, Fundación Instituto de Investigación Sanitaria de Santiago de Compostela FIDIS, 27003 Lugo, Spain; Cardiology Department, Hospital Universitario Lucus Augusti, 27003 Lugo, Spain; CardioHULA Research Group, Fundación Instituto de Investigación Sanitaria de Santiago de Compostela FIDIS, 27003 Lugo, Spain; Cardiology Department, Hospital Universitario Lucus Augusti, 27003 Lugo, Spain; CardioHULA Research Group, Fundación Instituto de Investigación Sanitaria de Santiago de Compostela FIDIS, 27003 Lugo, Spain

**Keywords:** Amyloidosis, Coronary sinus, Thrombosis, Arrhythmia, Multimodality imaging, Echocardiography, Case report

## Abstract

**Background:**

Amyloidosis can affect the heart, causing arrhythmia, thromboembolic events, and sudden cardiac death. Coronary sinus thrombosis is an uncommon though life-threatening condition which requires early identification and management.

**Case summary:**

A 72-year-old Caucasian man, who recovered from out-of-hospital cardiorespiratory arrest, was diagnosed with coronary sinus thrombosis using cardiac imaging techniques. He had no history of invasive procedures and was diagnosed with cardiac amyloidosis based on an extra-cardiac biopsy positive for light chain amyloid, with consistent clinical, echocardiographic, and magnetic resonance criteria.

**Discussion:**

A high frequency of intracardiac thrombosis is seen in amyloidosis. However, coronary sinus thrombosis is an uncommon complication. A multimodality imaging approach appears to be useful for the early diagnosis of coronary sinus thrombosis. The low specificity of the clinical signs, as well as the fast impairment of the patients, could result in fatal complications such as acute myocardial infarction, arrhythmia, and sudden death. Early screening, particularly in high-risk patients, as well as the use of early anticoagulant therapy, could reduce the associated morbidity and mortality.

**Learning points**

- A multimodality imaging approach may be useful for the early diagnosis of coronary sinus thrombosis, which is essential to achieve successful treatment outcomes.
- In the absence of invasive cardiac procedures, the presence of underlying prothrombotic disorders must be ruled out.
- Early screening, particularly in high-risk patients, as well as early anticoagulant therapy, could reduce the associated morbidity and mortality.

## Introduction

Amyloidosis is characterized by extracellular deposits of protein fibrils. Currently, over 98% of the cases of diagnosed cardiac amyloidosis are due to fibrils made of monoclonal immunoglobulin–derived light chains (AL amyloidosis) or of transthyretin (ATTR amyloidosis) both in its hereditary (ATTRv) and its acquired form (ATTRwt).^1^ Amyloid light chain amyloidosis has an approximate incidence of 12 cases per million inhabitants and year, with an estimated prevalence of 30–45 thousand cases in the USA and the European Union.^2^ Myocardial involvement entails a worse prognosis and can lead to heart failure with diastolic dysfunction of the left ventricle, arrhythmias, thromboembolic events, and even sudden cardiac death.^3,4^

Coronary sinus thrombosis is an uncommon condition, resulting in the formation of thrombi in the main venous drainage system of the heart.^5^ Most cases reported in the literature are related to invasive cardiac procedures, though they may be also associated with other prothrombotic diseases.^5,6^ The clinical manifestations are unspecific including chest pain, shortness of breath, or ischaemic findings in the electrocardiogram, which makes diagnosis very challenging. It has been associated with a high morbidity–mortality in patients, and also its clinical management is not clearly established.^5,6^ A study by Feng *et al*.^7^ based on 116 autopsies from patients with cardiac amyloidosis found 33% of hearts with intracardiac thromboses (four of them on coronary sinus valve), specially in AL patients, and associated with a significant number of fatal events. In patients with coronary sinus thrombosis, multimodality cardiac imaging play an pivotal role in the diagnosis process, improving the clinical management of this patients.^6,7^

## Summary figure

**Table:** 

Date	Event
**At admission**	The patient shows up after a recovered out-of-hospital cardiorespiratory arrest. The coronary angiography did not reveal angiographically significant lesions.
**1 day after admission**	The transthoracic and transoesophageal echocardiograms revealed a dilated coronary sinus with thrombosis inside. Treatment is started with intravenous sodium heparin.
**4 weeks after admission**	The magnetic resonance imaging performed confirmed left ventricular myocardial thickening and severe biatrial enlargement with thrombosis in multiple areas (coronary sinus and left atrial appendage).
**8 weeks after admission**	Cardiac scintigraphy revealed grade 1 cardiac uptake consistent with light chain amyloidosis.
**13 weeks after admission**	The control echocardiogram reveals resolution of the thrombosis.
**16 weeks after admission**	Oral connective tissue biopsy with diffuse deposit of amyloid substance with positive Congo red staining. The diagnosis of cardiac AL amyloidosis is established.

## Case report

A 72-year-old Caucasian man was evaluated after suffering a recovered out-of-hospital cardiorespiratory arrest. He has a history of high blood pressure, dyslipidaemia, anticoagulated atrial fibrillation with acenocoumarol, and previous ischaemic stroke.

**Figure 1:**
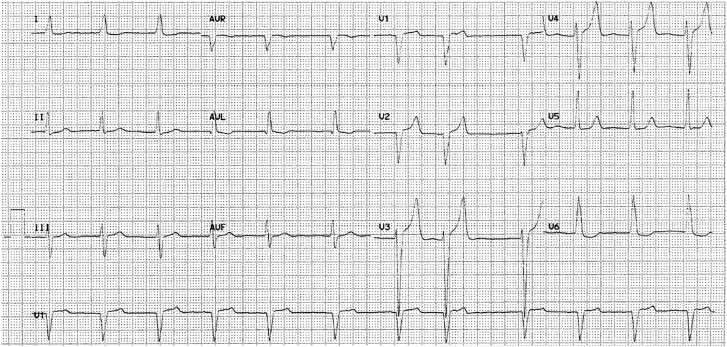
Electrocardiogram. The electrocardiogram shows atrial fibrillation with controlled ventricular response and left bundle block.

The patient was admitted to the intensive care unit (ICU) after cardiorespiratory arrest due to ventricular fibrillation recovered with three automatic defibrillator discharges. On admission to the ICU, he was haemodynamically stable, with no inotropic support, with orotracheal intubation (Glasgow Coma Scale 7), connected to assisted ventilation, and physical examination revealed jugular venous distension with left parasternal systolic murmur of grade 2/6. The electrocardiogram showed atrial fibrillation with left bundle block (*Figure 1*).

**Figure 2:**
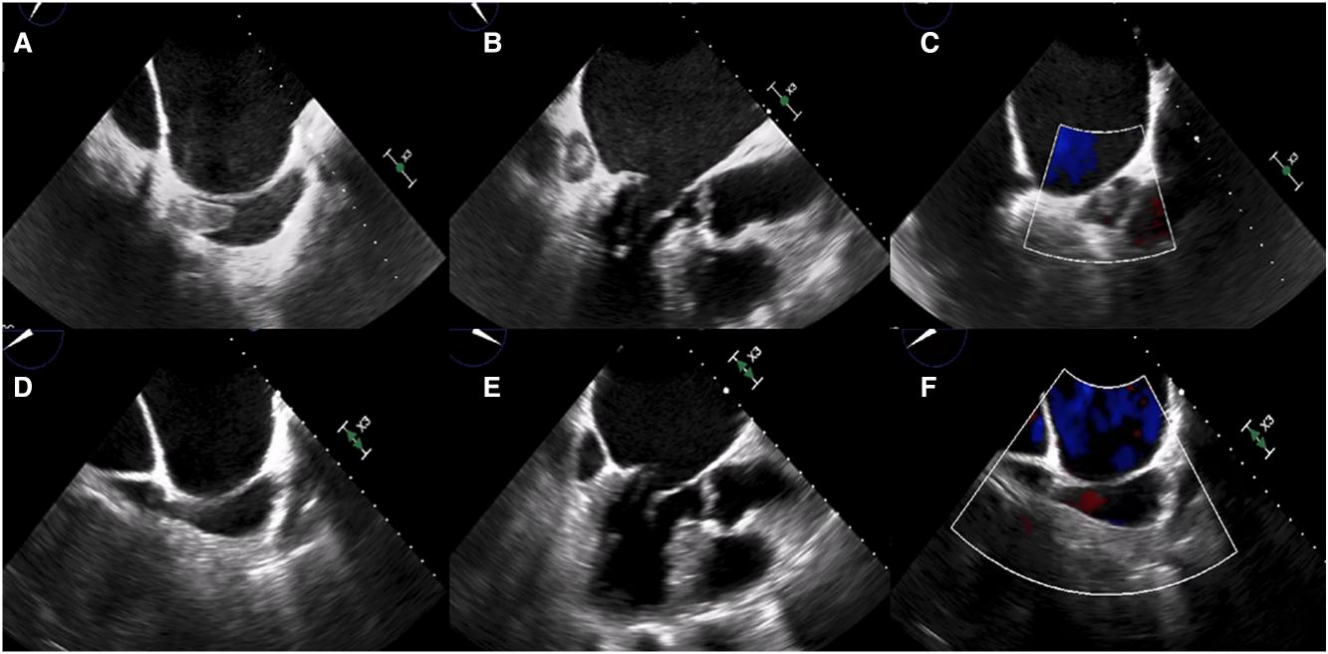
Transoesophageal echocardiogram. Transoesophageal echocardiogram showing thrombosis in the coronary sinus (*A–C*). New transoesophageal echocardiogram performed 4 weeks after admission that shows resolution of thrombosis with persistent residual sinus dilatation (*D–F*).

A first transthoracic and transoesophageal echocardiogram was performed at bedside which evidenced mild left ventricular hypertrophy with normal ejection fraction, severe biatrial enlargement, and estimated systolic pulmonary artery pressure of 50 mmHg. The most remarkable findings were dilated coronary sinus (25 mm) with an echodense non-calcified mass of large size inside (38 × 16 mm) close to the ostium, consistent with thrombus formation (*Figure 2A–C*; Supplementary material online, *Videos S1* and *S2*).

**Figure 3:**
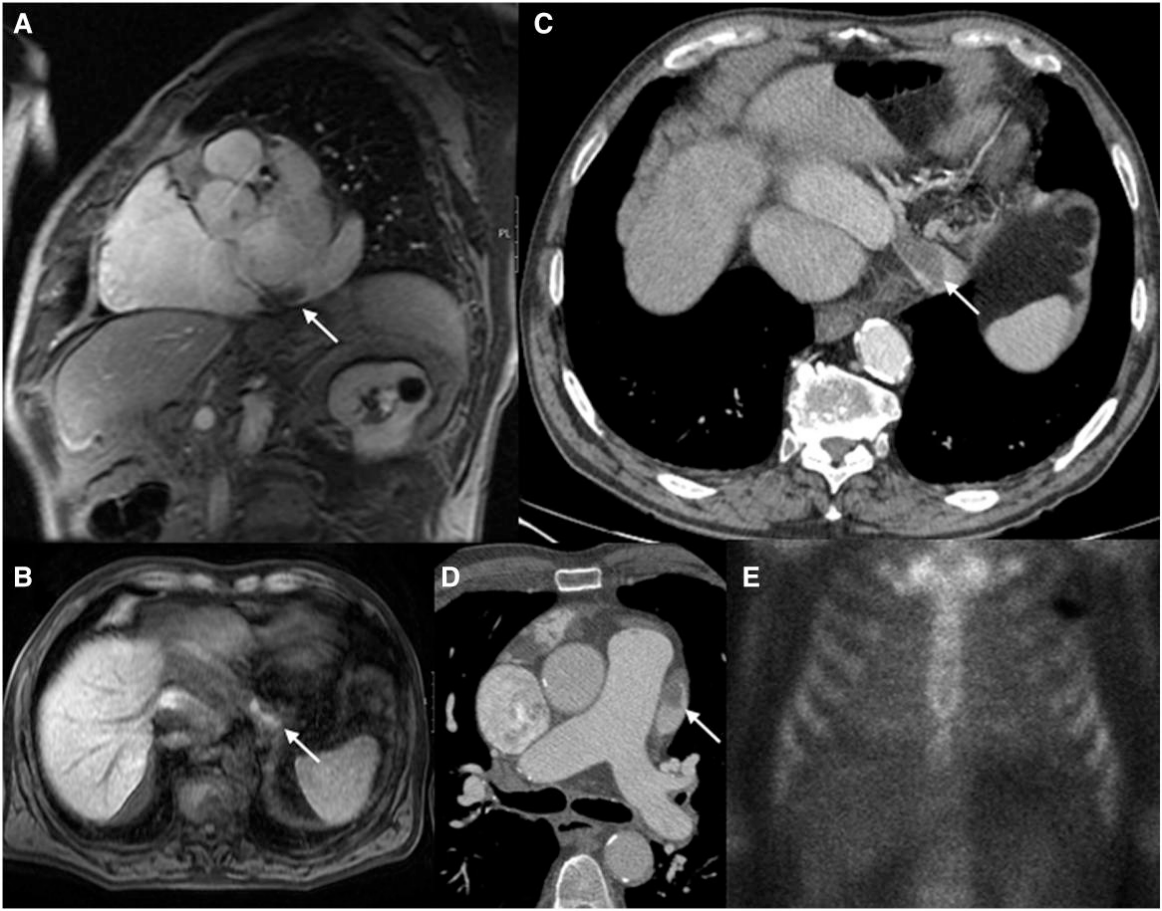
Cardiac magnetic resonance, computed tomography, and 99Tc-DPD scintigraphy. Dilatation of coronary sinus with thrombosis inside (arrow) in short-axis view (*A*); hyper-intensive view (arrow) in pre-contrast sequences in the coronary sinus consistent with early thrombus (*B*). Images of computed tomography showing coronary sinus thrombosis (arrow) in portal phase (*C*) and thrombosis (arrow) in left atrial appendage (*D*). Image of cardiac technetium-99m-labelled 3,3-diphosphono-1,2-propanodicarboxylic acid (99Tc-DPD) scintigraphy shows slight uptake (grade 1) of labelled disphosphonates in the myocardium with diffuse distribution (*E*).

The coronary angiography did not show significant coronary artery stenosis, and to be noted is only a dilated coronary sinus with no contrast wash-in. The magnetic resonance performed confirmed the left ventricular myocardial thickening (>12 mm) with severe biatrial enlargement and coronary sinus dilation with thrombus inside, in the oblique vein, vortex of the left atrial appendage, and left ventricle outflow tract (*Figure 3A–D* ).

Given the presence of intracardiac thrombosis, anticoagulant treatment with intravenous sodium heparin for 4 weeks was started. The clinical course in ICU was favourable and extubation was carried out after 24 h. The patient remained haemodynamically stable and was transferred to the hospitalization cardiology unit 72 h after admission. A new transoesophageal echocardiogram performed 4 weeks after admission confirmed the resolution of the coronary sinus thrombosis (*Figure 2D–F*). During his hospital stay, it was decided to implant an implantable cardioverter-defibrillator (ICD), and the patient was discharged once recovery was achieved.

**Figure 4:**
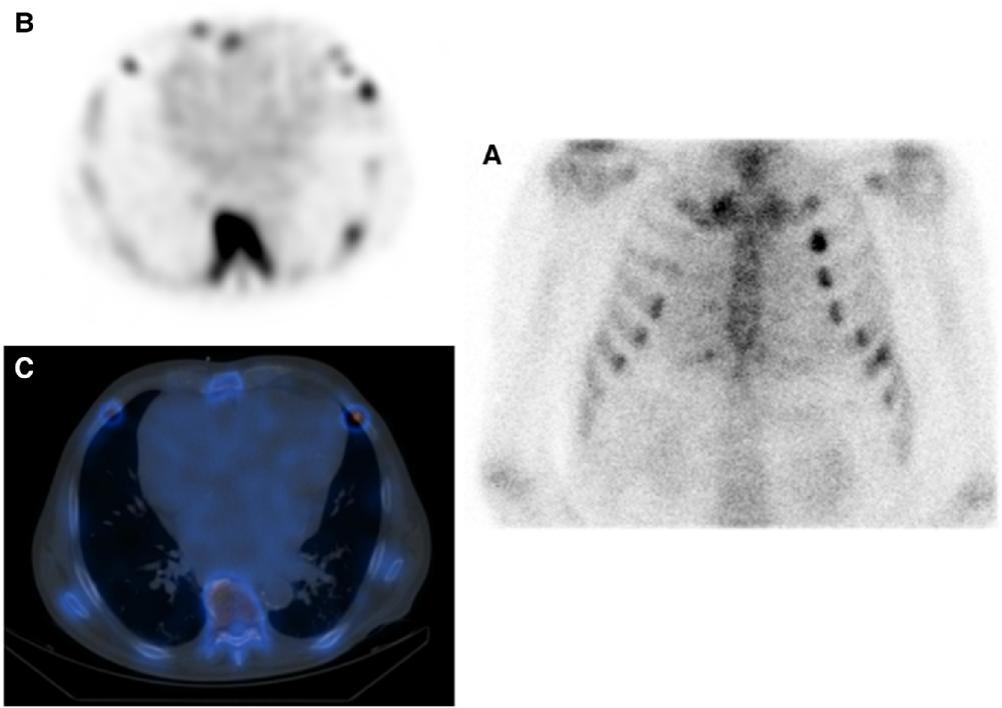
Cardiac scintigraphy and SPECT/CT. Cardiac scintigraphy evidenced grade 1 cardiac uptake consistent with light chain amyloidosis (*A* and *B*) and cardiac single photon emission computed tomography (SPECT/CT) showing cardiac involvement (*C*).

**Figure 5:**
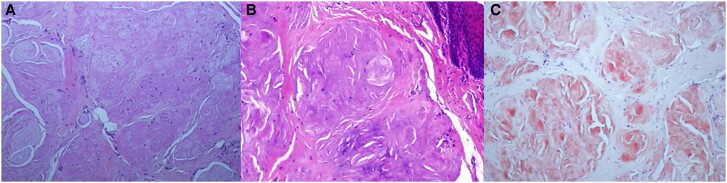
Tissue biopsy. Fragment of oral connective tissue with diffuse deposit of amyloid substance using haematoxylin-eosin stain (magnification ×200) (*A* and *B*) with positive Congo red staining and resistant to permanganate (magnification ×200) (*C*).

In the aetiological tests to rule out amyloidosis, screening for cancer and infectious diseases was performed, which proved to be negative. Cardiac Technetium-99–labelled 3,3-diphosphono-1,2-propanodicarboxylic acid (99Tc-DPD) scintigraphy evidenced grade 1 cardiac uptake consistent with AL amyloidosis (*Figure 3E* and *Figure 4A* and *B*). A cardiac single-photon emission computed tomography (SPECT/CT) was performed showing cardiac involvement (*Figure 4C*). The diagnosis was completed with an oral connective tissue biopsy positive for Congo red staining, with diffuse deposits of AL amyloid (*Figure 5*).

A multidisciplinary team carried out the treatment and follow-up. Due to high comorbidity and patient’s decision, a conservative management without chemotherapy was undertaken. Two years follow-up was favourable without ICD therapies, thromboembolic events, or hospitalization due to decompensated heart failure.

## Discussion

The coronary sinus is part of the venous drainage system of the heart and receives ∼60% of the venous return, which flows into the right atrium.^5,6^ Coronary sinus thrombosis is a rare, poorly recognized condition, due to the limited experience and the fast clinical impairment of the patients, which appears to underestimate further the reported prevalence and incidence.^5–7^

The mechanism is similar to the thrombosis, which occurs in other sites and results of the combination of venous stasis, changes in the coagulation profile, and lesion of the vessel wall. It is a complication subsequent to the endothelial lesion caused in invasive right heart procedures, although, less frequently, cases of spontaneous thrombosis have been described in relation to endocarditis, congenital defects, valvular lesions, atrial fibrillation, and hypercoagulability states.^5–7^

Acute coronary sinus thrombosis following invasive cardiac procedures is the most commonly reported.^8^ It generates an increase in intravascular and transcapillary pressure that decreases coronary artery blood flow, inducing complications such as acute myocardial infarction, arrhythmia, and even sudden death. A mechanism of venous and capillary engorgement also occurs, which can lead to cardiac effusion and tamponade.^5^ Chronic or incomplete occlusion can remain silent, due to an efficient collateral circulation, with occasional progression to complete occlusion or embolization of the thrombus.^8^ Given its low frequency and the poor specificity of the presenting symptoms, its diagnosis and treatment are challenging.

Coronary sinus dilation, with or without the presence of thrombus, can be detected by transthoracic echocardiography. However, due to its small size and the distance to the chest wall, sometimes it is difficult to obtain high-resolution images, so transoesophageal echocardiography plays an essential role.^9^ Computed tomography, magnetic resonance, and coronary angiography also provide relevant information. It appears that the multimodality imaging approach may be useful for the early diagnosis of this condition, essential to achieve successful treatment outcomes.^6^

The therapeutic management is not clearly defined and varies from patient to patient. Anticoagulant therapy is essential in therapeutic approach, both in stable and unstable patients. Although the clinical course after thrombectomy is unclear, cases of recovery of ventricular function have been reported.^10,11^

Long-term anticoagulant treatment with vitamin k antagonists or direct oral anticoagulants are safe and effective in these patients.^12^ The history and form of clinical presentation of our patient led to suspecting the existence of amyloidosis based on the presence of red flags.^1^ The past clinical history of patient included no family history of cardiovascular disease, sudden death or amyloidosis and without previous echocardiograms or other cardiovascular exams different that electrocardiograms. However, the patient had a series of personal history related to AL amyloidosis: macroglossia identified for the first time in an ear, nose, and throat evaluation due to hearing loss 2 years before admission; proteinuria and mild chronic kidney disease studied by nephrology; and chronic polyneuropathy diagnosed in a previous neurophysiological study.

The combination of extra-cardiac biopsy with AL amyloid deposit and consistent images in the ultrasounds, cardiac magnetic resonance, scintigraphy, and SPECT allowed to diagnose AL amyloidosis with cardiac involvement.^1^

The mechanism whereby intracardiac thrombosis occurs in patients with amyloidosis is not clear. Myocardial infiltration by amyloid fibrils leads to atrial dysfunction and enlargement, which causes blood stasis and risk of formation of thrombi.^12^ The endomyocardial damage, endothelial dysfunction, and hypercoagulability could also contribute.^7^

A study by Roberts *et al*.^13^ performed in 54 necropsies with cardiac amyloid deposits (49 of AL type) identified the presence of intracardiac thrombosis in 26% of the patients. Subsequently, Feng *et al*.^7^ reported, in patients with cardiac amyloidosis, a 33% of intracardiac thrombosis (coronary sinus thrombosis in 4 patients) based on 116 autopsies (55 of AL type). In this study, the AL group had significantly more intracardiac thrombosis (51% vs. 16%) associated with an extremely high risk of thromboembolism in the presence of associated atrial fibrillation.^7^

In cardiac amyloidosis, there is a high rate of intracardiac thrombosis. Early screening with multimodality cardiac imaging, as well as the use of early anticoagulant therapy, could reduce the associated morbidity and mortality in these patients.^7^

## Supplementary Material

ytae453_Supplementary_Data

## Data Availability

The data underlying this article are available in the article and in its online material.

## Lead author biography

Alejandro Manuel López Pena studied medicine at the University of Santiago de Compostela in Spain. He began his residency in cardiology in 2022. He is currently physician in residency in the Department of Cardiology at the Hospital Universitario Lucus Augusti in Lugo, Spain.

## Supplementary material

**Consent:** We obtained consent from the patient in accordance with COPE guidelines.

**Funding:** None declared.
